# UV-B Exposure Affects the Biosynthesis of Microcystin in Toxic *Microcystis aeruginosa* Cells and Its Degradation in the Extracellular Space

**DOI:** 10.3390/toxins7104238

**Published:** 2015-10-20

**Authors:** Zhen Yang, Fanxiang Kong

**Affiliations:** State Key Laboratory of Lake Science and Environment, Nanjing Institute of Geography and Limnology, Chinese Academy of Sciences, 73 East Beijing Road, Nanjing 210008, China; E-Mail: fxkong@niglas.ac.cn

**Keywords:** UV-B radiation, microcystin, gene transcription, degradation

## Abstract

Microcystins (MCs) are cyclic hepatotoxic heptapeptides produced by cyanobacteria that can be toxic to aquatic and terrestrial organisms. MC synthesis and degradation are thought to be influenced by several different physical and environmental parameters. In this study, the effects of different intensities of UV-B radiation on MC biosynthesis in *Microcystis* cells and on its extracellular degradation were investigated by mRNA analysis and degradation experiments. Exposure to UV-B at intensities of 1.02 and 1.45 W/m^2^ not only remarkably inhibited the growth of *Microcystis*, but also led to a decrease in the MC concentration. In addition, *mcyD* transcription was decreased under the same UV-B intensities. These results demonstrated that the effects of UV-B exposure on the biosynthesis of MCs in *Microcystis* cells could be attributed to the regulation of *mcy* gene transcription. Moreover, the MC concentration was decreased significantly after exposure to different intensities of UV-B radiation. Of the three MC variants (MC-LR, -RR and -YR, L, R and Y are abbreviations of leucine, arginine and tyrosine), MC-LR and MC-YR were sensitive to UV-B radiation, whereas MC-RR was not. In summary, our results showed that UV-B radiation had a negative effect on MC production in *Microcystis* cells and MC persistence in the extracellular space.

## 1. Introduction

Blooms of the cyanobacterium *Microcystis* sp. in eutrophic lakes, ponds and reservoirs have become severe environmental problems in many countries. Some *Microcystis* species are known to produce microcystins (MCs), which are cyclic hepatotoxic heptapeptides with over 85 natural structural variants that cause health problems in terrestrial and aquatic organisms [1,2]. MC levels in water bodies are mainly regulated by the following three factors: the biomass of the MC-producing cyanobacteria [3], MC production by toxigenic strains [4] and MC degradation or adsorption in water bodies [5].

As secondary metabolites, MCs are synthesized nonribosomally via a mixed polyketide synthase/nonribosomal peptide synthetase system termed MC synthetase [6]. MC synthetase is encoded by two transcribed operons in *M. aeruginosa*, *mcyABC* and *mcyDEFGHIJ*, from a central bidirectional promoter located between *mcyA* and *mcyD* [7]. It has been reported that the MC production of toxigenic strains is regulated by environmental factors, such as temperature [8], light intensity [9,10], nutrient concentrations or sources [11,12,13] and pH [14]. Some of these factors are involved in enhancing or repressing the expression of the *mcy* gene, leading to an increase or decrease in MC production, respectively. Kaebernick *et al.* [9] reported that light quality has a significant effect on the transcript levels of *mcyB* and *mcyD*, and Sevilla *et al.* [12] demonstrated that iron starvation causes an increase in *mcyD* transcription and MC levels. Moreover, it was also shown that stress conditions caused by nutrient deprivation clearly increased *mcyD* transcription and MC production [15].

All MC variants are named using the convention MC-XZ, where X and Z are two variable *L*-amino acids; X is commonly leucine (L), arginine (R), or tyrosine (Y); and Z is arginine (R) or alanine (A) [7]. The toxicity of MC is generally related to its amino acid composition. MC-LR, MC-YR and MC-RR are the three most common toxic variants, and of these, MC-LR is the most toxic [16]. MCs are extremely stable and resistant to chemical hydrolysis and oxidation at near-neutral pH due to their cyclic structures. In natural waters and in the dark, MCs may persist for months [17]. However, they can be oxidized by ozone and other strong oxidizing agents and photodegraded by solar irradiation near their absorption maximum [18]. In addition, bacteria in water bodies or cyanobacterial sludge, such as *Arthrobacter* sp. [19], *Ochrobactrum* sp. [20] and *Acinetobacter guillouiae* [21], are associated with high rates of MC biodegradation and have been considered to be useful in methods of MC elimination in the field.

Ozone and aerosols are the primary filters in the atmosphere that reduce damaging UV radiation before it reaches the Earth’s surface. However, because of the depletion of the stratospheric ozone layer that has occurred over the last century, the amount of solar UV-B reaching the Earth’s surface has increased remarkably since the 1980s [22]. UV-B radiation can penetrate to ecologically-significant depths in aquatic systems and exerts serious deleterious effects on phytoplankton [23,24,25]. Excessive buoyancy due to gas vacuole formation leads to the accumulation of *Microcystis* at the water surface when waves are not strong; thus, these bacteria are directly exposed to UV-B radiation. Several studies have demonstrated that UV-B radiation causes severe damage to *Microcystis*. Exposure to high-intensity UV-B can damage the acceptor side of Photosystem II (PSII) as well as genetic material and cytoskeletal elements, and it can induce the production of reactive oxidants and increase the sedimentation rate in *Microcystis* cells [26,27,28]. Interestingly, the non-MC-producing strains showed greater susceptibility to UV-B stress compared to MC-producing strains, which indicates that MCs play an important role in protecting cyanobacteria under stress conditions [28,29]. Conversely, UV-B exposure was shown to cause a decrease in MC levels in both the intracellular and extracellular environment of MC-producing *Microcystis* [29].

To investigate how the potential regulatory mechanisms triggered by UV-B radiation affect the intracellular and extracellular MC concentrations, the expression of the *mcy* gene in MC-producing *Microcystis* cells and MC degradation in the extracellular space were analyzed in this study upon exposure to different intensities of artificial UV-B radiation. The results will provide a more comprehensive view of the effect of UV-B radiation on the production of MCs and their fate.

## 2. Results

### 2.1. Cell Growth

**Figure 1:**
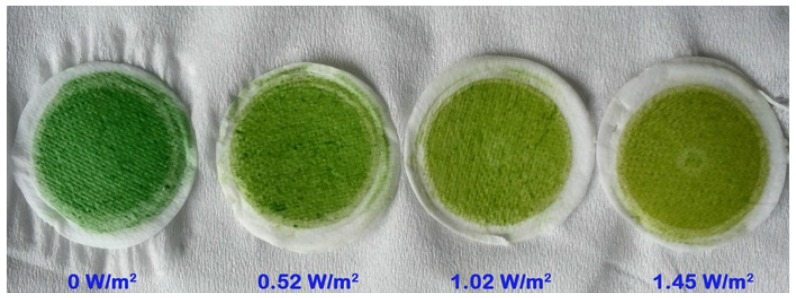
*M. aeruginosa* exposed to different intensities of UV-B radiation for 10 days.

**Figure 2:**
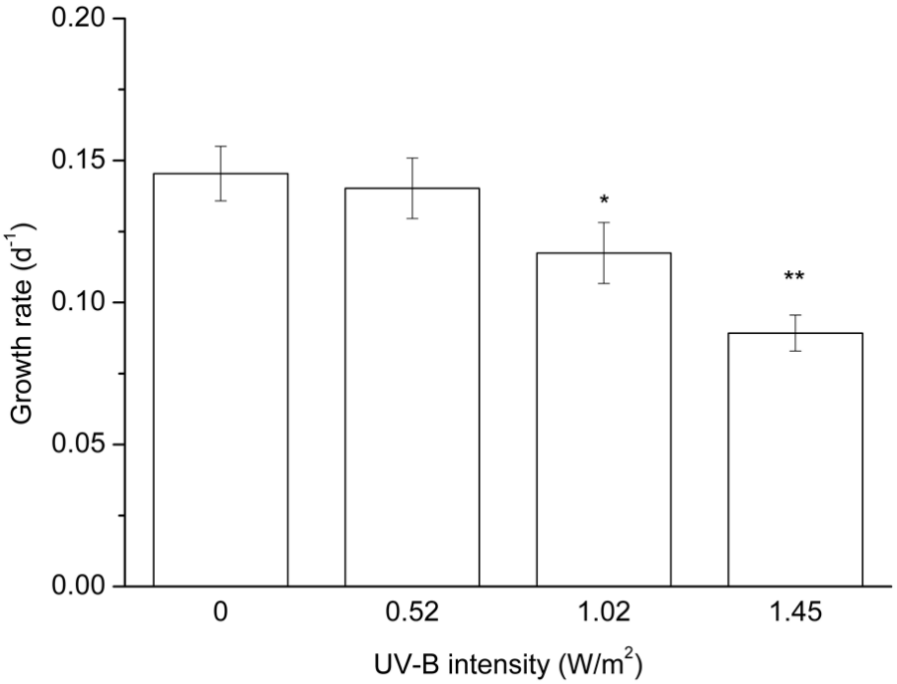
Growth rates of toxic *M. aeruginosa* exposed to different intensities of UV-B radiation for 10 days. The data are the mean ± SD (*n* = 3). * Statistically-significant difference of *p* < 0.05 when compared to the control (0 W/m^2^); ** statistically-significant difference of *p* < 0.01.

Exposure of *M. aeruginosa* to different intensities of UV-B radiation caused varying degrees of growth inhibition*.* The algal cells became brown in color following several days of exposure to UV-B radiation (Figure 1), and the cell growth rates showed negative dose-dependent correlations with UV-B radiation (Figure 2). *M. aeruginosa* cells exposed to 1.45 and 1.02 W/m^2^ of UV-B radiation showed growth rates of 0.09 and 0.12, respectively, after 10 days of culture. These rates were significantly lower compared to the growth rates of cells that had not been exposed to UV-B.

### 2.2. Intracellular and Extracellular MC Concentrations

**Figure 3:**
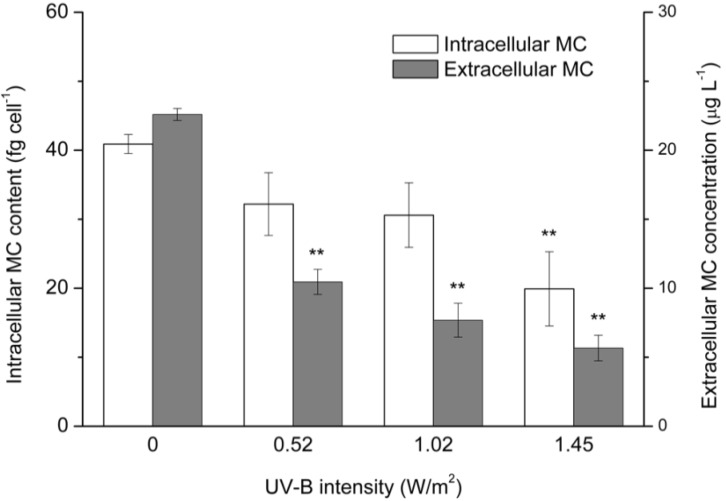
Intracellular and extracellular microcystin (MC) concentrations of toxic *M. aeruginosa* exposed to different intensities of UV-B radiation for 10 days. The data are the mean ± SD (*n* = 3). ** Statistically-significant difference of *p* < 0.01 when compared to the control (0 W/m^2^).

UV-B radiation negatively affected both the intracellular and extracellular MC levels (Figure 3). An MC concentration of 40.93 fg·cell^−1^ was detected in toxic *M. aeruginosa* cells in the absence of UV-B exposure. The MC levels deceased to 32.22 and 30.64 fg·cell^−1^ following exposure to 0.52 and 1.02 W/m^2^ of UV-B radiation, respectively, for 10 days. The intracellular MC levels in toxic *M. aeruginosa* cells exposed to 1.45 W/m^2^ of UV-B radiation were less than half of those observed in cells that had not been exposed to UV-B. The negative effect of UV-B radiation on extracellular MCs was more remarkable. The extracellular MC concentrations were 10.46, 7.68 and 5.67 μg·L^−1^ following exposure to 0.52, 1.02 and 1.45 W/m^2^ of UV-B radiation, respectively. These levels were significantly lower than those measured in the absence of UV-B exposure.

### 2.3. mcy Gene Expression

**Figure 4:**
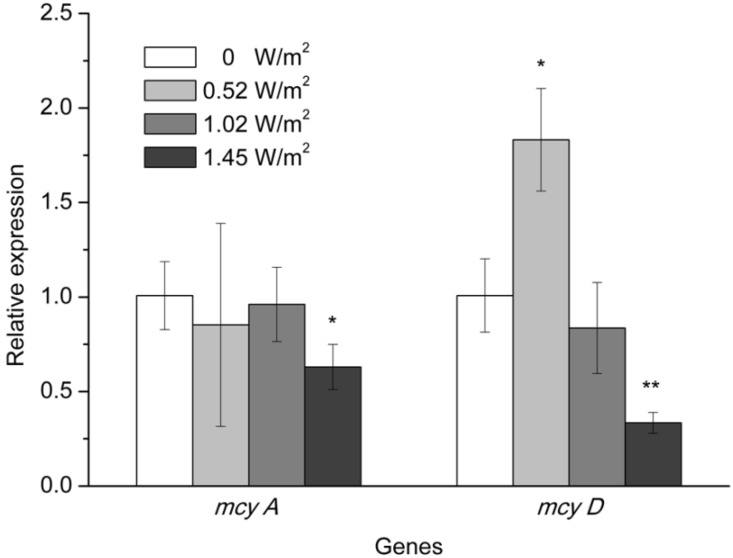
Relative expression level of *mcy* genes in toxic *M. aeruginosa* exposed to different intensities of UV-B radiation for 10 days. The data are the mean ± SD (*n* = 3). * Statistically-significant difference of *p* < 0.05 when compared to the control (0 W/m^2^); ** statistically-significant difference of *p* < 0.01.

The RNeasy Plant MiniKit used in this study was very efficient for RNA extraction from *M. aeruginosa* cells. The extracted RNA concentrations were >100 ng/μL, which was sufficient for reverse transcription. The expression of the toxin genes fluctuated with changes in UV-B radiation intensity (Figure 4). The relative expression level of the *mcyA* gene under 1.45 W/m^2^ of UV-B radiation was significantly lower than that observed in the absence of UV-B exposure. Exposure to lower intensities of UV-B (0.52 and 1.02 W/m^2^) did not result in dramatic changes in *mcyA* gene expression. The effect of UV-B exposure on *mcyD* gene expression was remarkable. Exposure to lower intensities of UV-B radiation had a positive effect on *mcyD* expression. UV-B radiation at an intensity of 0.52 W/m^2^ increased *mcyD* expression by 1.83-fold compared to its level in unexposed algal cells. In contrast, in the treatments with 1.02 and 1.45 W/m^2^ of UV-B radiation, the relative expression level of the *mcyD* gene decreased to approximately 83% and 30% of that measured in the unexposed algal cells, respectively.

### 2.4. MC Degradation

**Figure 5:**
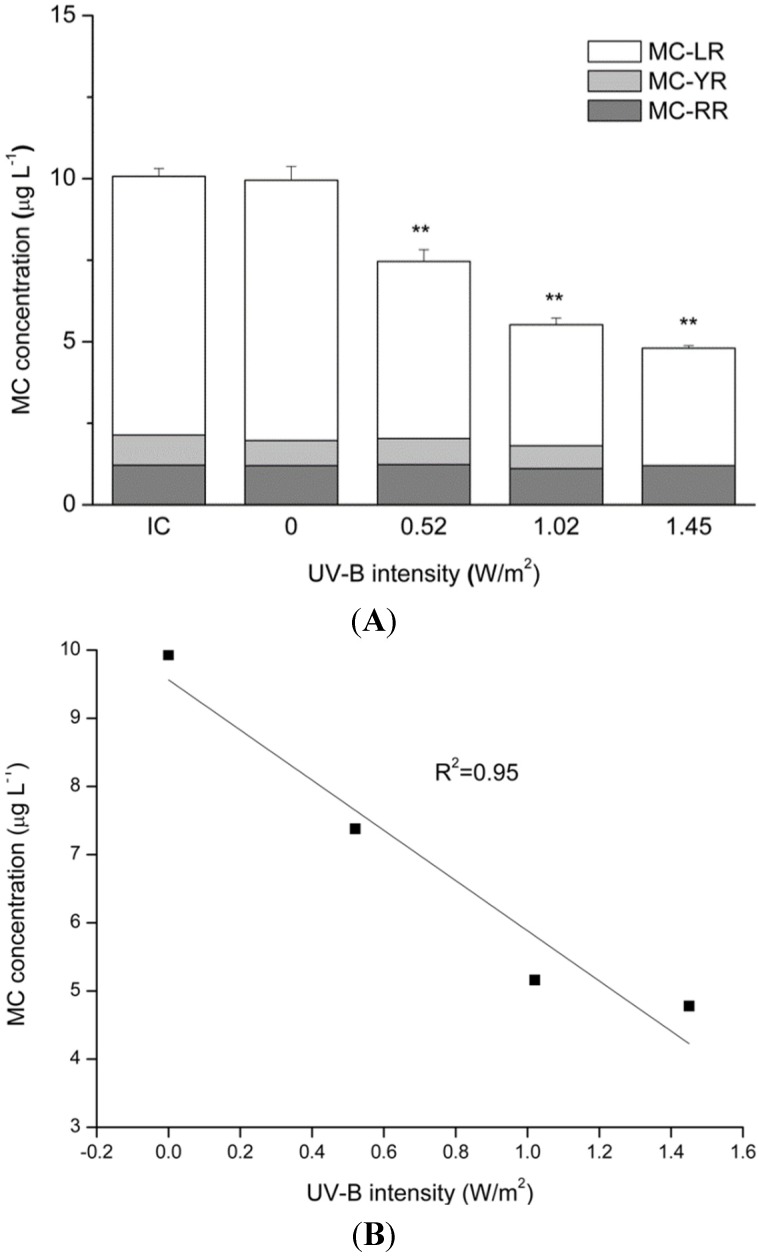
(**A**) Concentrations of three MC variants and total MCs under different intensities of UV-B radiation for 24 h. IC: initial concentration treatments and control (0 W/m^2^) before the experiment. The data are the mean ± SD (*n* = 3). ** Statistically-significant difference of *p* < 0.01 when compared to the initial concentration. (**B**) Linear relationship of UV-B radiation intensity and MC concentration.

UV-B exposure caused considerable degradation of MCs (Figure 5A). No significant change was found in the MC concentration in the absence of UV-B exposure. In contrast, there was a strong linear relationship (*R*^2^ = 0.95) between UV-B radiation intensity and MC concentration (Figure 5B). The MC concentration decreased from 10.07 μg·L^−1^ at the beginning of the experiment to 7.26, 5.35 and 4.80 μg·L^−1^ following exposure to 0.52, 1.02 and 1.45 W/m^2^ of UV-B radiation, respectively. Of the three MC variants, MC-LR and MC-YR were sensitive to UV-B radiation. The MC-LR and MC-YR concentrations decreased considerably following exposure to UV-B for 24 h, while the change in MC-RR was small.

## 3. Discussion

In our previous study, we reported that both intracellular and extracellular MC levels decreased markedly when MC-producing *Microcystis* was exposed to UV-B radiation. In the present study, we performed further investigation using mRNA analysis and degradation experiments. For the first time, we were able to show that exposure to high-intensity UV-B decreased the transcription of *mcyD*, whereas low-intensity UV-B stimulated the expression of *mcyD*. Additionally, we observed that MCs in the extracellular space were photodegraded after several hours of UV-B exposure. This is the first report describing the potential regulatory mechanisms of MC biosynthesis and degradation under UV-B exposure.

UV-B radiation-induced stress has been reported in *Microcystis* in several previous studies. Exposure to high-intensity UV-B inhibits the growth of this alga by damaging the acceptor side of PSII [26], inhibiting photosynthetic activities [29] and inducing the production of reactive oxidants [27]. Although the synthesis of both mycosporine-like amino acids (MAAs), which absorb UV radiation, and carotene, which counteracts reactive oxidants, may allow *Microcystis* to cope with low-intensity UV-B radiation, damage caused by high-intensity UV-B radiation cannot be discounted [30]. Growth inhibition at a rate of approximately 20% and 30% was respectively observed under treatment with 1.02 and 1.45 W/m^2^ of UV-B radiation for 4 h per day (Figure 2). This result indicated that the stress level was increased with increasing intensities of UV-B radiation in *Microcystis*. During a sunny day in the spring or summer, the UV-B radiation level on the Earth’s surface can be maintained at 1.4 to 1.6 W/m^2^ for several hours [31]. Therefore, the stress that was induced in *Microcystis* as a result of UV-B radiation exposure in the current study can actually occur under natural conditions, especially at water surfaces.

HPLC analysis in this study revealed that UV-B radiation affected the intercellular and extracellular levels of MCs in *Microcystis* (Figure 3). MCs are synthesized nonribosomally by enzyme complexes containing nonribosomal peptide synthetase (NRPS) and polyketide synthase (PKS). Genes encoding the enzyme complex are part of a large gene cluster containing at least two operons, *mcyABC* (peptide synthetase) and *mcyDEFGHIJ* (hybrid polyketide-peptide synthetase) [7]. The *mcy* gene has been proposed to be regulated by a complex network involving nutrient limitation, dual transcriptional regulation and stress-inducing factors [32,33]. Several batch culture experiments have demonstrated that *mcyD* transcription correlates to MC content when the MC-producing *Microcystis* cell was cultivated under different iron or nutrient conditions [12,34]. Furthermore, in this study, a decreased *mcyD* transcript and MC content simultaneously was found under a high intensity of UV-B exposure. We may infer from this result that depressed *mcyD* transcript by high UV-B stress is a trigger for the decreased MC content in *Microcystis* cell.

In this study, we used 16S rRNA as the single endogenous reference gene to normalize the expression changes of target genes. Because differences in RNA expression may occur in cells at different times and after various types of experimental manipulation, many authors have proposed the use of two or more housekeeping genes to normalize RNA levels [35,36]. However, in previous studies, 16S rRNA showed stable expression under different light and nutrient conditions in *Microcystis* [9,34], and it was previously used as a reference or housekeeping gene to normalize expression changes in target genes [12,15,37].

The level of *mcyD* transcription was significantly reduced by exposure to UV-B radiation at intensities ranging from 0.52 to 1.45 W/m^2^ in a dose-dependent manner. Similar trends were found by Qian *et al.* [37], who reported that *mcyD* exhibits a dose-dependent expression pattern in response to H_2_O_2_ and CuSO_4_ stress. It has been demonstrated that high light would result in increased *mcyD* transcript levels at certain threshold intensities [9]. *Microcystis* cells in the control of this study were cultivated under cool white fluorescent light at an intensity of 40 μmol quanta·m^−2^·s^−1^. At the low UV-B radiation treatment (0.52 W/m^2^), the light intensity reaching the culture increased by about 30% and resulted in increased *mcyD* transcription. On the other hand, recent studies have suggested that MCs may play an important role in the increased fitness of toxic cyanobacterial strains under high light and UV radiation stress [28,29,38]. The protein-modulating function of this secondary metabolite protects cyanobacteria against oxidative stress [39]. In addition, the increase in extracellular polysaccharides induced by MCs facilitates *Microcystis* colony formation [40]. A colony with a diameter of several micrometers will exhibit increased sunscreen efficiency because of the self-shading provided by several layers of cells; thus, sunscreen efficiency is positively correlated with colony size. Therefore, the increased transcription of the *mcyD* gene observed under low-intensity UV-B radiation in this study may have been an adaptive strategy to cope with UV-B radiation-induced stress. In contrast, stress caused by high-intensity UV-B radiation would be disruptive and irreversible, not only resulting in growth inhibition, but also leading to damage to the photosynthetic apparatus and causing downregulation of *mcyD* gene transcription, which requires an active photosynthetic electron transfer chain [4].

Compared to its effect on *mcyD*, the effect of UV-B radiation on *mcyA* transcription was less severe. *mcyA* and *mcyD* belong to two operons and have bidirectional promoters. In addition, the alternative transcription start points of *mcyA* and *mcyD* are different and appear to be light dependent [41]. *mcyD* transcription requires an active photosynthetic electron transfer chain and could be affected by oxidative stress, which indicate that this gene is highly sensitive to light [4]. Kaebernick *et al.* [9] found that the *mcyD* transcript level was increased under high light intensity and red light, whereas it was decreased under light with a shorter wavelength (blue light). Additionally, these authors postulated that the negative effect of UV-B exposure on *mcyD* transcription may be attributed to the UV-B-induced inhibition of the photosynthetic activities in *Microcystis* cells. Because the polyketide synthase encoded by the *mcyD* cluster is involved in the production of the β-amino acid Adda, which is responsible for the toxicity of MCs [7], decreased *mcyD* transcription is more important for decreasing the quantity of substrate available for MC synthesis.

Because the wavelengths of UV-C radiation (200 nm to 280 nm) are near the absorption maximum of MCs (238 nm), this type of shortwave radiation can quickly induce structural changes in the MC molecule, typically leading to the formation of conformational isomers, such as 6(*Z*)-Adda-microcystin-LR [42]. When applied in combination with photocatalysis, TiO_2_ and ZnO, which cause multiple hydroxylations and eliminate labile peptide residues of the MC molecule, can achieve nearly complete removal of these toxins within several minutes [43]. The wavelengths of UV-B radiation are near those of UV-C radiation (ranging from 280 to 315 nm), and in this study, UV-B radiation caused considerable degradation of MCs after a long period of exposure (Figure 5). This result demonstrated that in addition to the inhibition of intracellular MC synthesis, MC degradation by UV-B radiation also contributed to the decreased extracellular MC concentration shown in Figure 3. Moreover, this result supports the findings of previous studies [44], which have described the potential importance of indirect photolytic degradation in the elimination of MCs by plotting residual MC concentration against the cumulative UV radiation energy of sunlight in natural water.

In addition to the toxin concentration, changes in toxicity have also been concerning to researchers studying photodegradation, because these changes can be used to determine health risk. The stability of MCs may depend on the sensitivities of their amino acids to different environmental conditions, Therefore, MC variants showed different stability in some published data, as they contain variable L-amino acids [45]. For example, the chlorine reaction rates for MC-YR, MC-RR and MC-LR differ and are ranked in the following order: MC-YR > MC-RR > MC-LR [46]. However, under UV-visible light, MC-LR and MC-YR show greater sensitivity than MC-RR to photocatalyst materials [47]. Similarly, UV-B exposure in our study led to substantial degradation of MC-LR and MC-YR, but did not significantly affect MC-RR. This finding could indicate that photodegradation by UV-B radiation actually affects the Adda amino acid and may diminish the toxicity of MCs to terrestrial and water-based organisms.

## 4. Experimental Section

### 4.1. Experimental Design

The toxic cyanobacterium *Microcystis aeruginosa* (Kützing) Kützing FACHB 915 was obtained from the Institute of Hydrobiology, Chinese Academy of Sciences and grown in Blue-Green Medium 11 (BG11) culture medium at 25 °C under cool white fluorescent light at an intensity of 40 μmol quanta·m^−2^·s^−1^ with a light:dark cycle of 12:12 h. Once this strain had reached the exponential stage of growth, it was inoculated into BG11 medium and cultivated in Petri dish bottoms (diameter: 15 cm, height: 2.5 cm) with a thin polyethylene membrane seal that transmitted more than 96.1% of the UV-B radiation to prevent contamination. UV-B radiation at effective intensities of 0 (control), 0.52, 1.02 and 1.45 W/m^2^ was provided for 4 h per day (from 10:00 to 14:00) by placing the dishes under UV-B lamps (Philips, Aachen, Germany; TL20W/01RS) at different distances. The experiments were performed for 10 days at 25 °C under a 12:12 light:dark cycle with light provided at an intensity of 40 μmol quanta·m^−2^·s^−1^. Each treatment group and control included three replications.

Samples were obtained after 10 days of culture and divided into subsamples to determine cell counts (Cell Analyzer, Muse, Darmstadt, Germany), as well as extracellular and intracellular MC levels. In addition, a 50-mL sample was taken after 10 days of culture to analyze the effect of UV-B radiation on *mcy* gene expression.

For the toxin-producing algal cell culture, extracellular MC concentration depended both on intracellular MC content and their stability in different extracellular environments. It is not accurate to assess the degradation of UV-B exposures to MCs by analysis of the MC concentration in the supernatant of cells cultures after UV-B exposures directly, because their concentration was also affected by MCs released from intracellular environment during experiment. Therefore, the degradation of extracellular MCs by UV-B radiation was assessed as follows: 1.2 L of toxic *M. aeruginosa* culture were filtered through glass microfiber filters (Whatman GF/C, Maidstone, England; pore size:1.2 μm) to filter out the algal cell and obtain a filtrate that contains an amount of MCs. One hundred milliliters of each filtrate were added to a Petri dish bottom with a thin polyethylene membrane seal and exposed to UV-B radiation at intensities of 0, 0.52, 1.02 and 1.45 W/m^2^ for 24 h, as described above.

### 4.2. Gene Expression Analysis

Fifty-milliliter aliquots of *M. aeruginosa* were centrifuged at 4000× *g* for 5 min. The supernatant was poured off, and the resulting cell pellet was resuspended with 1.5 mL of the remaining media and centrifuged again at 4000× *g* for 5 min. Finally, the cell pellets were immediately flash-frozen in liquid nitrogen and stored at −80 °C.

Total RNA was extracted using an RNeasy Plant MiniKit (QIAGEN, Hilden, Germany) according to the manufacturer’s recommendations. To disrupt the cell pellets completely, samples were moved into the lysing matrix tubes (containing 1.4-mm ceramic spheres, 0.1-mm silica spheres and one 4-mm glass bead with a purple cap, MP Biomedicals, Santa Ana, CA, USA) with RLT buffer and homogenized in a FastPrep-24 (MP Biomedicals) at Speed 6 for 30 s, and the spheres and glass beads were removed by centrifugation at 10,000× *g* for 2 min. Total RNA extracted from *M. aeruginosa* cells was re-suspended in 20 μL of Diethy pyrocarbonate (DEPC)-H_2_O. The integrity of RNA was assessed on a 1% agarose gel, and the RNA concentration was determined by using a spectrophotometer (Thermo, Waltham, MA, USA; Nano-Drop 2000) to measure the absorbance at 260 nm. RNA purity was assessed by calculating the A_260_/A_280_ ratio. Prior to reverse transcription, genomic DNA contamination of the RNA sample was eliminated using gDNA wipeout buffer and verified via conventional PCR with *mcyA*, *mcyD* and 16S rRNA primer pairs (Table 1), followed by 1% agarose gel electrophoresis. To prepare cDNA, 0.5 μg of total RNA was reverse transcribed using a Quanti Tect Reverse Transcription Kit (QIAGEN, Hilden, Germany) according to the manufacturer’s instructions.

The transcription of *mcyA* and *mcyD* was detected by real-time PCR using reverse-transcribed cDNA, and 16S rRNA was used as a housekeeping gene to normalize the expression changes. All of the reactions were performed in a volume of 25 μL containing 12.5 μL of 2× SYBR Green Real-time PCR Master Mix (TaKaRa, Shiga, Japan), 2.0 pmol each primer and 1 μL of cDNA from the standard strain or the sample to be analyzed. The reaction volumes were adjusted to a final volume of 25 μL with sterile ultra-pure water. The assay was performed with a Mastercycler Realplex4 (Eppendorf, Hamburg, Germany) equipped with Mastercycler Realplex software. Amplification was conducted as follows: an initial preheating step was performed for 2 min at 95 °C, followed by 40 cycles of 95 °C for 20 s, 58 °C for 30 s and 72 °C for 20 s (for 16S rRNA) or 40 cycles of 95 °C for 20 s, 56 °C for 30 s and 72 °C for 30 s (for *mcyA* and *mcyD*). The melting temperatures for the real-time PCR products were determined using the manufacturer’s software.

**Table 1:** Primer sets used in real-time PCR in this study.

Target Gene	Primers	Sequence (5′-3′)	Reference
*mcyA*	MSF MSF-2R	ATCCAGCAGTTGAGCAAGC GCCGATGTTTGGCTGTAAAT	[48]
*mcyD*	F2	GGTTCGCCTGGTCAAAGTAA	[9]
	R2	CCTCGCTAAAGAAGGGTTGA	
16S rRNA	184F	GCCGCRAGGTGAAAMCTAA	[49]
	431R	AATCCAAARACCTTCCTCCC	

The relative expression ratios were calculated according to the 2^−ΔΔC*t*^ method [50], which is based on the differences among the mean C*_t_* values of the treatment groups with normalization to the expression of the endogenous reference gene, 16S rRNA.

### 4.3. MCs Extraction and Analysis

The dynamics of the extracellular and intracellular MCs of the toxic strain were analyzed in the monoculture experiment. Aliquots of 20 mL were filtered through glass microfiber filters (Whatman GF/C; pore size: 1.2 μm). The filtrates were enriched with octadecylsilyl (ODS) cartridges and subsequently eluted with 10 mL of methanol solution (containing 0.1% trifluoroacetic acid). After the eluates were evaporated under N_2_, the residues were redissolved with 100% methanol and stored at −20 °C until the extracellular MC concentration was analyzed. To measure the intracellular MC concentration, 3 mL of 75% (*v/v*) aqueous methanol were added to a centrifuge tube containing a filter, sonicated for 10 min to disrupt the algal cells and centrifuged at 5000 rpm. Each sample was extracted three times. The supernatants were evaporated under N_2_, redissolved with 100% methanol and stored at −20 °C until analysis. MCs were analyzed by reverse-phase high-performance liquid chromatography (Agilent, Agilent, Palo Alto, CA, USA; Agilent1200) with photodiode array detection according to Wiedner *et al.* [51]. The total MC concentration was quantified as the sum of all MC peaks using standards for the three most common toxic variants: MC-LR, MC-RR and MC-YR (Sigma, Roedermark, Germany).

### 4.4. Data Analyses

The specific growth rates (μ d^−1^) of the *M. aeruginosa* cells were calculated according to the following equation: μ = ln(N*_t_*/N_0_)/*t*, where N*_0_* and N*_t_* are the initial cell density and the cell density after incubation for *t* days, respectively. Data of growth rate, MC concentration and *mcy* gene relative expression are presented as the mean ± 1 SD and were analyzed using SPSS 16.0. Significant differences between control and treated samples were determined by one-way ANOVA followed by a *t*-test. The linear fit analysis was conducted to determine the relationship of UV-B radiation intensity and MC concentration using Origin 8.

## 5. Conclusions

The present study shows that UV-B exposure leads to decreases in intracellular and extracellular MC concentrations in toxic *M. aeruginosa* cells. mRNA analysis showed that *mcyD* transcript levels were decreased under exposure to UV-B at an intensity of 1.02 and 1.45 W/m^2^, which may have been related to the decrease in the intracellular MC concentration. Moreover, the concentration of MCs (especially MC-LR and MC-YR) was decreased significantly after exposure to different intensities of UV-B radiation, demonstrating that UV-B exposure causes considerable degradation of MCs. These results indicate that UV-B radiation has a major impact on the MC level in cyanobacterial blooms.
